# Epidemiology of neonatal early-onset sepsis in a geographically diverse Australian health district 2006-2016

**DOI:** 10.1371/journal.pone.0214298

**Published:** 2019-04-08

**Authors:** Kathryn Braye, Maralyn Foureur, Koert de Waal, Mark Jones, Elise Putt, John Ferguson

**Affiliations:** 1 Faculty of Health, University of Technology, Sydney, New South Wales, Australia; 2 Hunter New England Health, Newcastle, New South Wales, Australia; 3 School of Nursing and Midwifery, University of Newcastle, Newcastle, New South Wales, Australia; 4 Department of Neonatology, John Hunter Hospital, Newcastle, New South Wales, Australia; 5 School of Biomedical Sciences and Pharmacy, University of Newcastle, Newcastle, New South Wales, Australia; 6 Hunter Medical Research Institute, Newcastle, New South Wales, Australia; 7 New South Wales Health Pathology, Newcastle, New South Wales, Australia; Monash University, AUSTRALIA

## Abstract

**Aim:**

To describe the epidemiology of EOS including blood culture utilisation, across a large and geographically diverse Australian health district.

**Background:**

Sepsis in the first three days of life remains a leading cause of death and morbidity. In high-income countries, group B *Streptococcus* (GBS) and *Escherichia coli (E*. *coli)* have dominated as causes of EOS for five decades.

**Method:**

An 11-year retrospective cohort study to determine the epidemiology of EOS. Incidence rates were calculated per 1000 live births. Logistic regression with linear temporal trend and covariates for potential effect modifiers were employed. Blood culture utilisation was determined by examining the rate of babies undergoing blood culture within 72 hours of birth.

**Results:**

Among 93,584 live born babies, 65 had confirmed EOS (0.69/1000 live births); 22 term, 43 preterm. **Across the 4 largest birth units, the proportion of babies having blood culture within 72 hours of birth varied from 1.9–5.1% for term and 21–35% for preterm babies.** The annual change in the EOS rate was significant, OR 0.91 (95% CI, 0.84 to 0.99, p = 0.03). Group B *Streptococcus* was the most common cause of EOS in term neonates at 0.35/1000 live births (95% CI, 0.07–0.63) in 2006 and 0.1/1000 live births (95% CI, 0–0.2) in 2016. *Escherichia coli* was the most common cause in preterm babies at 3.4/1000 (95% CI, 0.11–6.76) in 2006 reducing significantly to 1.35/1000 live births (95% CI, -0.07–2.78) by 2016.

**Conclusions:**

*Escherichia coli* and GBS were the most common causes of EOS in preterm and term babies respectively. Rates of all cause term and preterm EOS declined significantly as did preterm sepsis due to *E*. *coli*. While rate of sepsis due to early-onset GBS declined, this did not reach significance. Given the high proportion of preterm babies undergoing blood culture, it is unlikely that any EOS events were missed.

## Introduction and Background

Neonatal EOS refers to culture-proven bloodstream and/or central nervous system infection occurring in live born neonates soon after birth. It remains a significant cause of infant morbidity and mortality in both high and low-income countries [1]. Most events occur in the first 48 hours of life [2]. Based on varying definitions, recent reported estimated incidences of culture proven EOS range from 0.01 to 0.53/1000 live births in Europe [3], 0.77/1000 in the USA [4, 5] to 0.83/1000 in Australia [6]. Recorded mortality has been as high as 30% in high-income and 60% in low-income countries[3] but has reduced in high income jurisdictions in recent years to around 10% of all babies with EOS [3, 7]. Preterm babies continue to suffer a much higher mortality than term infants [8]. Neonatal EOS is almost always due to pathogenic microorganisms acquired from the mother, either during or preceding birth [2, 5]. Infection may be trans-placental (haematogenous) but is more commonly thought to occur by an ascending route from the mother’s genitourinary tract [9].

### Risk factors for early-onset sepsis

Perinatal maternal risk factors reported to be associated with EOS include recto-vaginal GBS colonisation, rupture of membranes (ROM) ≥18 hours, prematurity, and intrapartum fever [3]. Recently authors have questioned the value of including fever as a surrogate for chorioamnionitis[10]. However currently, maternal fever (≥38°) remains a risk factor for EOS in most jurisdictions [8, 11–13]. Other risk factors include bacteriuria in the index pregnancy and having a previous child diagnosed with early-onset group B streptococcal infection (EOGBS) [11].

### Pathogens

Early-onset sepsis due to *E*. *coli* is associated with high rates of mortality and morbidity, especially in preterm newborns [5]. The rate of multiple resistant *E*. *coli* has been described as stable overall but increasing in very low birth weight neonates (<1.500g) [14]. In term babies, population-based neonatal infection surveillance studies demonstrate that GBS remains the pathogen most frequently associated with term EOS [6, 15–17].

### Early-onset group B streptococcal sepsis

Whilst there is a decline in early-onset GBS (EOGBS) sepsis whenever any maternal intrapartum antibiotic prophylactic (IAP) is provided [18] and others have noted evidence for IAP to be strong [6, 19], the most recent Cochrane review on the effectiveness of IAP concludes that whilst IAP appeared to reduce EOGBS, this may be due to bias. The Cochrane reviewers found a high risk of bias for one or more key domains in the methodology and execution of the studies included in their analysis [20]. Therefore, Cochrane states there is a lack of evidence from well designed and conducted trials to recommend IAP to reduce neonatal EOGBS. More recently we undertook a broader, integrative review and concluded the evidence that the incidence of EOGBS can be reduced by widespread administration of maternal IAP is not robust [20].

## Aim

To describe the epidemiology of EOS including blood culture utilisation, across a large and geographically diverse Australian health district.

## Materials and methods

A retrospective cohort study design was employed using data from a cohort of live born babies and their mothers birthing in the Hunter New England local health district (HNELHD) in New South Wales (NSW), Australia over the period 2006–2016.

### Study setting and population

New South Wales is the largest and most populous state in Australia covering a region of 131,785 square kilometres, equivalent to the size of England. In 2008 the state was divided into 15 local health districts (LHDs). The HNELHD is geographically and socially diverse. As of 2016, the health district had 873,741 residents. Aboriginal and Torres Strait Islander people account for 4.0% (equating to 21% of NSW’s Indigenous population) and 19% of residents were born overseas [21]. The health district spans 25 local government areas and is the only regional health district in NSW with a metropolitan hospital and level three neonatal intensive care unit (NICU), an along-side birth centre and a freestanding birth centre nearby. In total, these centres support around 4000 births per year. The district also includes a mix of several large regional hospitals providing care for 500 to 1500 births per year through to 11 rural units servicing a small percentage of people located in more remote settings supporting <250 births per year.

Our study population included women from the original Hunter area birthing in 2006 to 2007. From 2008 onwards when HNELHD was formed, the cohort of women increased to include the 16 units mentioned above. The study population included women and babies of all risk categories, birthing within all publicly funded maternity services in the HNELHD including hospital, birth centre and planned births at home. From 2006 onwards women in the Hunter area/HNELHD were offered universal screening for GBS risk.

In this paper the term “pregnancies” better describes the total cohort of women as one woman could have been pregnant more than once during the study period, however, the term “women” represents all women and each of her pregnancies resulting in a live birth(s) from 2006 to 2016.

### Blood culture utilisation

To evaluate EOS surveillance intensity, data from 2013 to 2016 were analysed to determine the proportion of babies from whom at least one blood culture was collected during the first 72 hours of life stratified by gestation (term or preterm).

### Demographics

**Fig 1:**
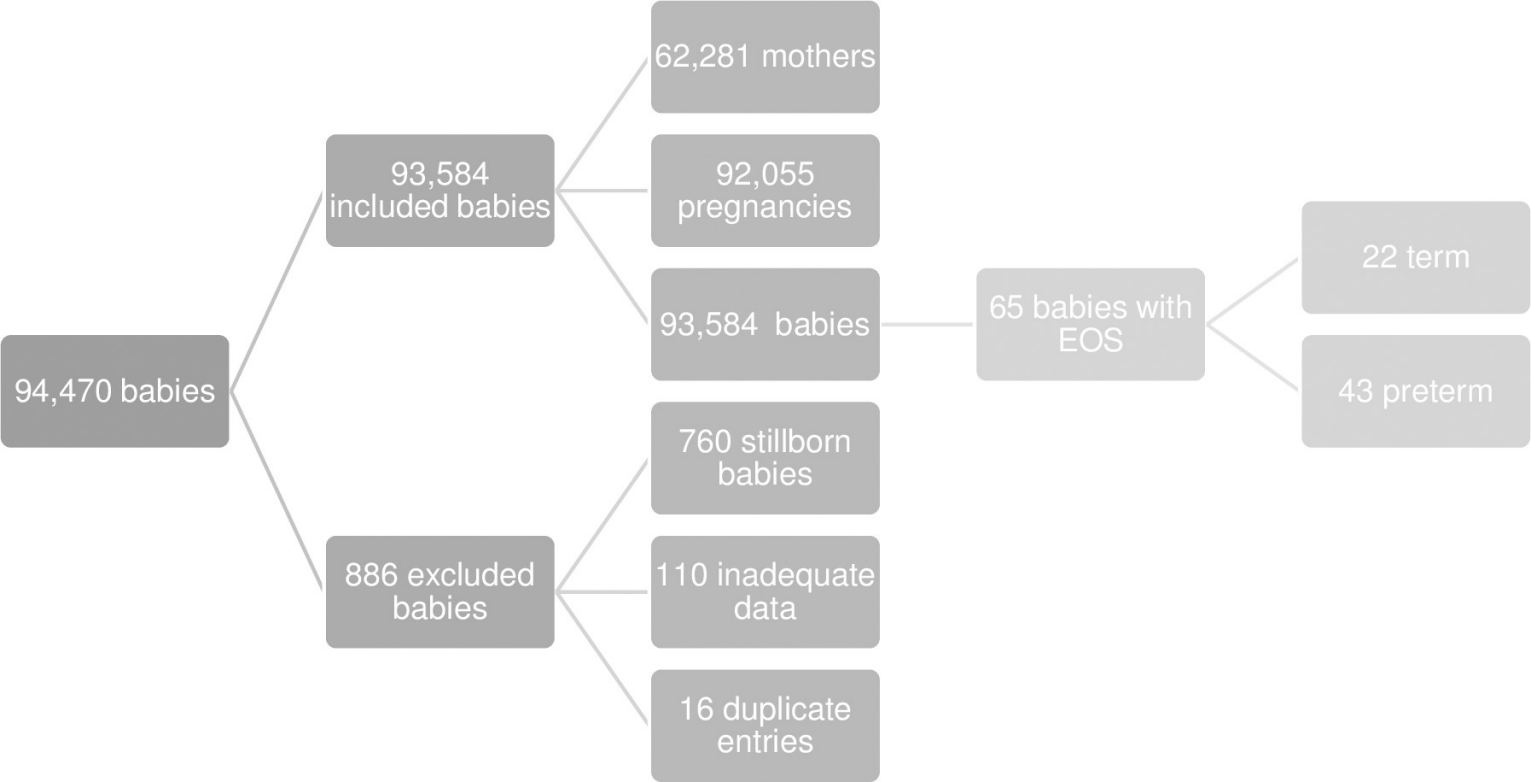
Inclusions and exclusions.

Inclusion criteria comprised live born infants and their mothers, born in the Hunter area 2006 to 2007 and expanded to include the newly formed HNELHD between 1^st^ January 2008 and 31^st^ December 2016. Stillborn babies, duplicate entries and entries with inadequate data were excluded Fig 1.

Information was obtained from medical records and the maternity ObstetriX database. ObstetriX (changed to e. Maternity in 2017) is a state wide surveillance system providing point-of-care data collection across antenatal, intrapartum, and immediate postnatal periods. Clinicians contribute information soon after birth. Local health district data custodians maintain the database.

We collected demographic data for mothers and babies, antenatal and intrapartum risk factors for EOS together with the neonatal hospital course and short-term neonatal outcomes. Maternal GBS colonisation, prematurity, and rupture of membranes (ROM) ≥18 hours, plus maternal age (age is categorical including ≤20 years versus all others), were collected and used in analysis. While history of maternal GBS bacteriuria and a previous child with EOGBS are both GBS risk factors, considered in a decision to offer IAP, we were unable to obtain information on either. Data on rates of fever were poorly transcribed into the database and therefore were not included as a risk factor for sepsis in our analysis.

### Microbiological cultures

Results of culture positive blood and cerebrospinal fluid (CSF) specimens were accessed from the publicly funded pathology service provider (Auslab) and three of four private pathology providers. Early-onset sepsis events were identified from laboratory data, when significant isolates (bacteria or fungi) were obtained from blood culture and/or CSF collected within the first 72 hours of life. Cultures yielding ≥3 bacterial species or a potential contaminant e.g. coagulase negative *Staphylococci*, as specified by Schrag and colleagues, 2016 were assumed to have been contaminated and were therefore excluded [5].

### Definition of early-onset sepsis

Researchers use a range of time frames to define EOS ranging from 48 hours to 7 days post birth [11]. The definition used here is neonatal sepsis arising within 72 hours of birth in accord with the definition used by the UK National institute for clinical care excellence (NICE) [3, 22].

### Morbidity and mortality

Admission and short-term morbidity were reported as serious or not serious. Serious morbidity was defined as the need for significant respiratory or circulatory support requiring neonatal intensive care and/or encephalopathy or seizures. It was not possible to assess long-term morbidity. Live status of each baby with an EOS event as of December 2017 was derived from the HNELHD patient demographics system, which is linked to NSW death registration data.

### Prevention of group B streptococcal sepsis

Between 2006 and 2008, starting with the metropolitan unit in HNELHD, a program of GBS screening for women reaching ≥35 weeks’ gestation was commenced. Mothers with recto/vaginal GBS colonisation or certain other risk factors for EOGBS (as documented above) are offered IAP in labour, or at rupture of membranes if this occurs first in order to reduce the likelihood of EOGBS.

### Ethics approval

The study was approved by the HNELHD Human research ethics committee (HREC) on 05/05/2016 with variation applied for and granted 09/03/2018 16/05/18/5/5.05 SSA. LNRSSA/16/HNE/225 and The University of Technology Sydney (HREC): No. 2014000115. This publication adheres to the provision of privacy and confidentiality of patient data and clinical information, including NSW health records and information privacy act, 2002. The purpose of our study was to describe the epidemiology of EOS rather than individual events; therefore consent from individuals was not required. All data was de-identified and password protected.

### Statistical analysis

Descriptive statistics on sample characteristics are provided. Incidence rates were calculated as cases per 1000 live births per year with t-tests being used for continuous variables. Chi-squared tests were used for proportions and Wilcoxon rank sum tests for comparisons of medians. P-values >0.05 suggest a statistically detectable difference between groups, which may or may not be clinically relevant and/or meaningful. We explored temporal trends in background all-cause EOS sepsis in the individual birth data by using logistic regression with a linear temporal trend and covariates for potential effect modifiers.

Potential effect modifiers used were positive maternal GBS recto-vaginal colonisation, prematurity (<37 weeks gestation), rupture of membranes (ROM) ≥18 hours and maternal age (<20 years versus all others). We provide estimates of odds ratios, graphical visualisation, probabilities of events and the associated 95% confidence intervals and p-values. All models were checked for calibration and discrimination and we use a conventional significance level of 0.05 throughout.

## Results

### Study population

After exclusions, the study population included 62,281 women who had 92,055 pregnancies. These women gave birth to 93,584 babies. There was an increase in the birthing population during and after 2008 as the area restructured and HNELHD was formed. Ninety-eight per cent of live born babies (90,510) were singletons and 8,165 (8.9%) of the pregnancies were preterm resulting in 9,146 (9.8%) preterm live born babies. Eight per cent of women (9,336) identified as Aboriginal or Torres Strait Islander (ASTI).

Table 1 provides a descriptive statistical summary of the data. It reports various characteristics of our sample overall, stratified by EOS and No EOS. The p-values correspond to statistical comparisons between the No EOS and EOS strata. Table 1 suggests statistically significant differences between the No EOS and EOS groups for term versus preterm babies, type of pregnancy (singleton or other), ATSI mothers, rupture of membranes >18 hours and pregnancies with positive maternal GBS colonisation. These variables with the exception of the type of pregnancy (singleton versus other) and mothers that are ATSI are recognised risk factors for EOGBS Table 1A & 1B. These results suggest that risk factors for EOS appear in our sample.

**Table 1:** 1a & 1b. Sample characteristics of women, their pregnancies, and babies.

**Table 1a. Women**	**Value**	**Overall (%)**	**No EOS (%)**	**EOS (%)**	**P value**
Number of pregnancies		92055	91990 (99.9)	65 (0.1)	
Type of pregnancy	Singleton	90510 (98)	90453 (98)	57 (88)	<0.001
	Multiple	1545 (2)	1537 (2)	8 (12.3)	
Term pregnancy (%)	Term	83890 (91)	83868 (91)	22 (34)	<0.001
	Preterm	8165 (9)	8122 (9)	43 (66)	
Median maternal age [IQR]		28 [24 to 32]	28 [24 to 32]	28 [24 to 32]	0.682
Aboriginal/Torres Strait Islander	Yes	9336 (10)	9330 (10)	6 (9)	<0.001
	No	82590 (90)	82533 (90)	57 (88)	
	Not stated	103 (0.1)	103 (0.1)	0 (0)	
	Missing	26 (0)	24 (0)	2 (3)	
Rupture of membranes ≥ = 18	Yes	8881 (10)	8847 (10)	34 (52)	<0.001
	No	83129 (90)	83100 (90)	29 (45)	
	Missing	45 (0)	43 (0)	2 (0)	
GBS result (total pregnancies)	Positive	13288 (14)	13278 (14)	10 (15)	0.028
	Negative	48236 (53)	48212 (52)	24 (37)	
	Unknown	30531 (33)	30500 (33)	31 (48)	
Alcohol consumption	1 to < 5	2416 (3)	2415 (3)	1 (1.5)	0.002
	1+daily	236 (0.3)	236 (0.3)	0 (0)	
	5+daily	216 (0.2)	216 (0.2)	0 (0)	
	None	88406 (96)	88344 (96)	62 (95)	
	Unknown	522 (0.6)	522 (0.6)	0 (0)	
	Missing	259 (0.3)	257 (0.3)	2 (3)	
Smoking at booking	Yes	19143 (21)	19130 (21)	13 (20)	<0.001
	No	72469 (79)	72419 (79)	50 (77)	
	Unknown	184 (0.2)	184 (0.2)	0 (0)	
	Missing	17 (0)	17 (0)	0 (0)	
Prior pregnancy	Yes	66648 (72)	66606 (72)	42 (65)	0.369
	No	25390 (28)	25367 (28)	23 (35)	
	Missing	17 (0)	17 (0)	0 (0)	
**Table 1b. Babies**					
All babies		93584	93519 (99.9)	65 (0.1)	
Singleton	Yes	90510 (97)	90453 (97)	57 (88)	
Term	Yes	84438 (90)	84416 (90)	22 (34)	<0.001
	No	9146 (10)	9103 (10)	43 (66)	
Mean birth weight (SD)		3360 (641)	3360 (640)	2217 (1135)	<0.001
Median gestational age [IQR])		40 [38, 41]	40 [38, 41]	33 [29, 39]	<0.001

Legend: EOS = early-onset sepsis, [IQR] = interquartile range, ASTI = Aboriginal or Torres Strait Islander

1 to <5- = one drink daily and not more than 5 at one sitting, eligible = a pregnancy ≥ 35 weeks gestation, ROM = rupture of membranes, GBS = group B streptococcus

### Early-onset sepsis

**Fig 2:**
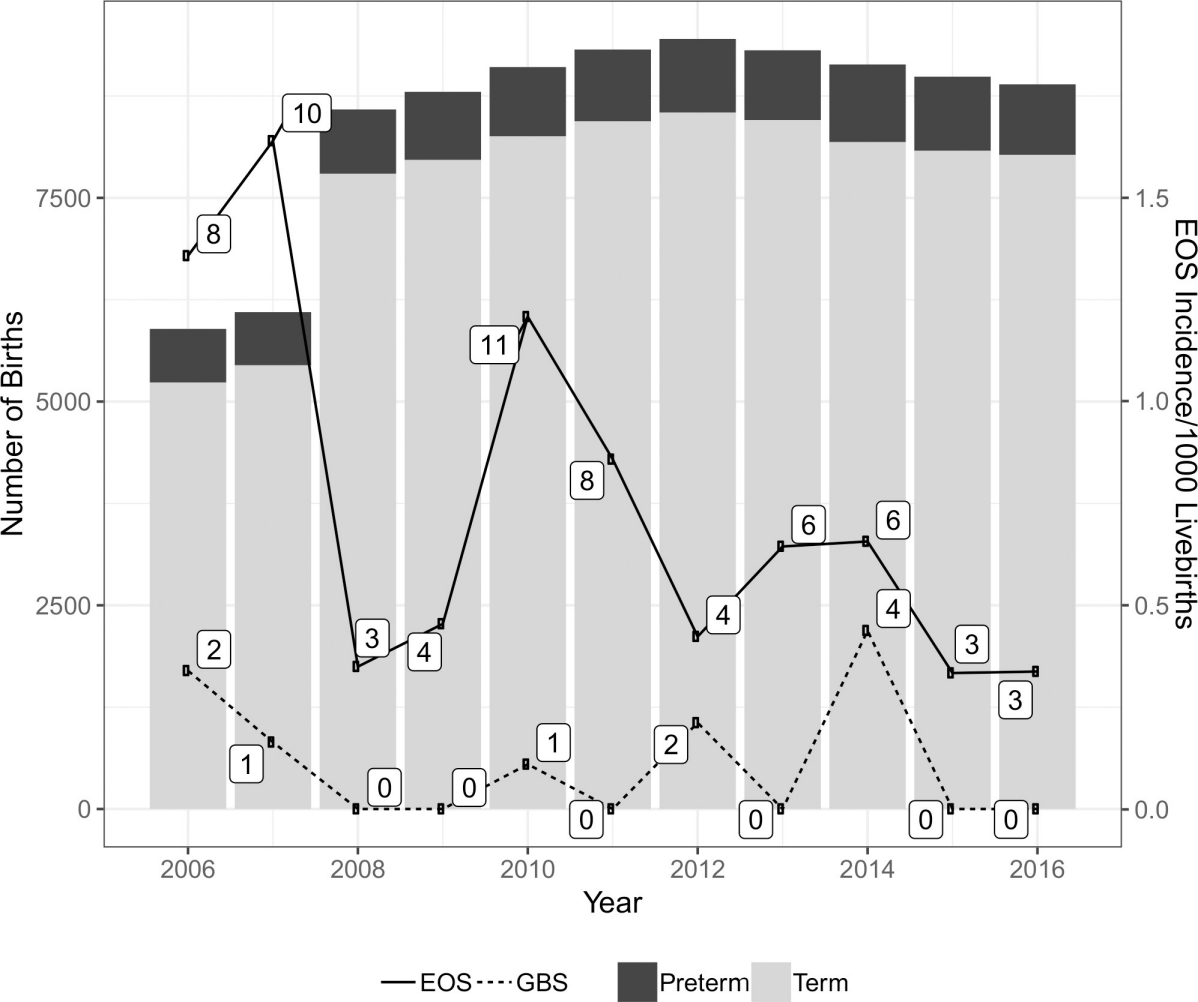
Early-onset sepsis events per 1000 live births.

In total, 65 culture-proven EOS events were identified Fig 2. Over 80% of EOS was evident within 48 hours of birth 82, and 88% for term and preterm groups respectively. Most, but not all, babies subsequently diagnosed with EOS were born at the metropolitan unit 50/65 (77%). The annual change in the background rate of all-cause EOS was significant at the 0.05 level with a multiplicative change in the odds of EOS reducing by approximately 9% per year over the study period as indicated by an odds ratio of 0.91 (95% CI, 0.84 to 0.99, p = 0.03). Six women who identified as ATSI mothers had babies with EOS Table 1A & 1B.

#### Term babies with early-onset sepsis

Twenty-two, (34%) of the 65 babies with EOS were term gestation. Baseline (2006) incidence of EOS in term infants was estimated with adjustment for gestation, birth weight, maternal GBS carriage, ROM ≥18 hours and maternal age to be approximately 0.5 (95% CI, 0.02 to 0.97)/1000 live births. By 2016, the rate was 0.19 (95% CI, 0 to 0.4).

#### Preterm babies with early-onset sepsis

**Fig 3:**
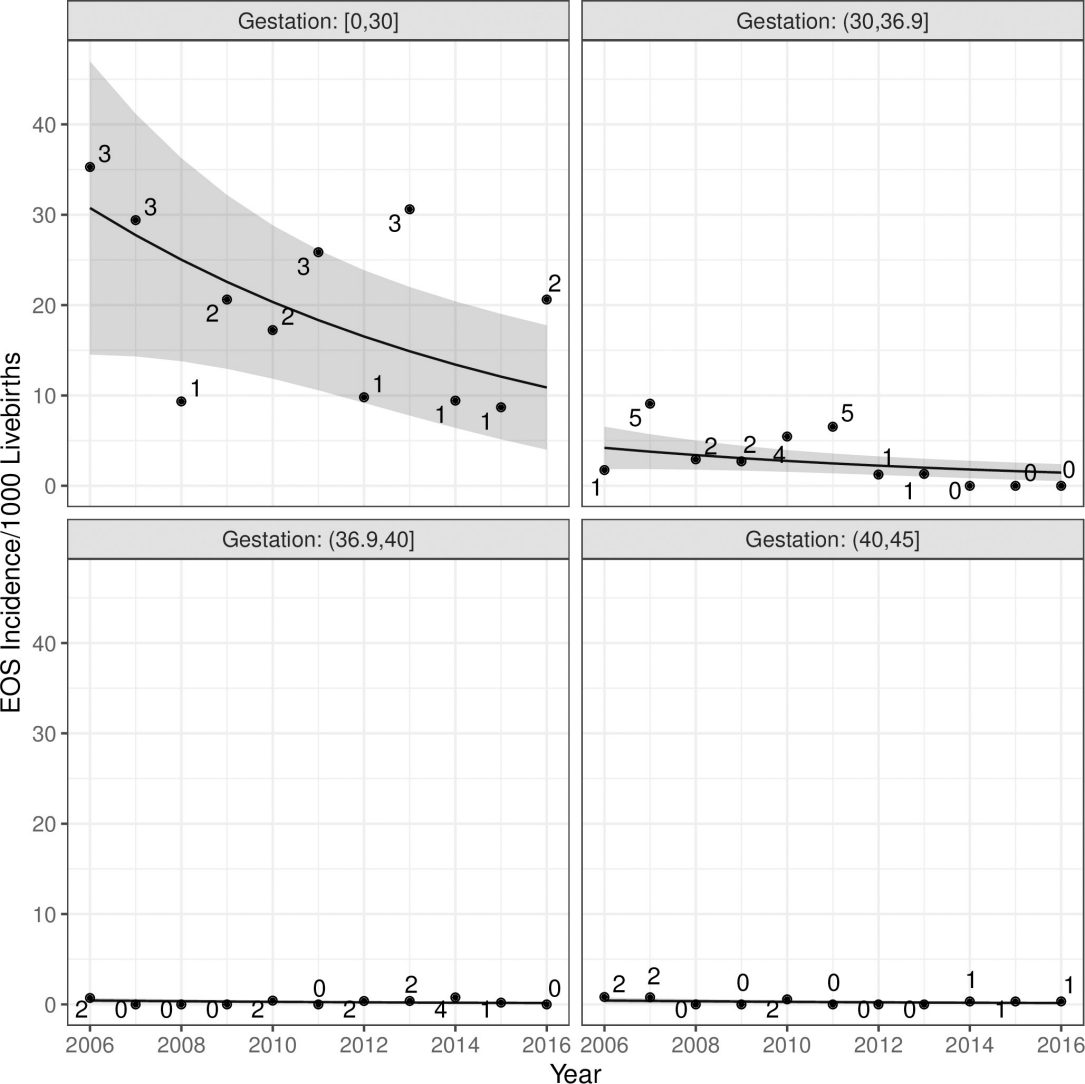
Early-onset sepsis events by gestation.

Forty-three babies who developed EOS were preterm. Baseline incidence of preterm EOS was estimated with adjustment for gestation, birth weight, maternal GBS colonisation, ROM ≥18 hours and maternal age to be approximately 22/1000 live births (95% CI, 1.3 to 42.7) in babies with gestations <30 weeks and 3.4/1000 live births (95% CI, 0.1 to 6.8) in babies with gestations 30 to 36 weeks and 6 days. By 2016, the respective rates had fallen significantly to 8.8/1000 live births <30 weeks (95% CI, -0.5 to 18) and 1.4/1000 live births between 30 and 36 and 6 days (95% CI, -0.1 to 2.8) Fig 3.

### EOS surveillance intensity 2013–2016

Across the 4 largest birth units, the proportion of babies having at least one blood culture within 72 hours of birth varied from 1.9 to 5.1% for term and 21 to 35% for preterm babies with large year-to-year variation. Across the smaller units that do not care for preterm babies, the proportion of babies cultured was 1.1% or less, with a mean of 0.7%.

### Diagnosis

All EOS events were diagnosed by blood culture. Forty-one (63%) babies also underwent lumbar puncture; 11/41 (27%) term and 30/41 (73%) preterm. Lumbar punctures were taken within two days of a positive blood culture in 31/41 (76%) cases. Of the 41 lumbar puncture tests taken, four (10%) were positive and results were in accord with the blood culture.

Gram-negative species predominated significantly in preterm EOS events, 24/43 (55%) and (4/23) 17% in term babies (p = 0.006); *E*. *coli* and other *Enterobacteriaceae* were the leading causes in 20/43 (45%) of the preterm cohort Table 2. The rate of EOS due to *E*. *coli* over the study period was 0.19/1000 live births (n = 18); 0.02/1000 live births (n = 2) in the term cohort and 1.75/1000 live births (n = 16) in the preterm cohort.

Gram-positive species were responsible for most EOS cases in term babies 18/22 (82%) versus 18/43 (42%) in preterm babies Table 2. Group B *Streptococcus* constituted almost half of EOS events in term babies at 10/22 (45%). The overall rate of EOGBS was 0.19/1000 (n = 18) live births.

**Table 2:** Term and preterm early-onset sepsis by organism.

Fungi	Term	Preterm	Total
*Candida glabrata*		1	**1**
**Gram negative bacteria**			
*Acinetobacter baumannii* complex	1		**1**
Escherichia coli*	**2**	**16**	**18**
*Haemophilus influenzae*		4	**4**
Klebsiella pneumoniae*		1	**1**
Morganella morganii*		1	**1**
*Pseudomonas aeruginosa*		1	**1**
*Salmonella* species*	1		**1**
Serratia marcescens*		1	**1**
**Gram positive bacteria**			
*Enterococcus faecalis*	2		**2**
*Enterococcus faecium*	1		**1**
***Streptococcus agalactiae* (GBS)**	**10**	**8**	**18**
*Streptococcus pneumoniae*	2	4	**6**
*Streptococcus* species (other)	2	4	**6**
*Staphylococcus aureus* (methicillin-susceptible)	1	2	**3**
**Total**	**22**	**43**	**65**

*These species are from the *Enterobacteriaceae* family.

No events due to methicillin-resistant *Staphylococcus aureus* or vancomycin-resistant enterococci were detected. Of the events due to *E*. *coli*, 67% were ampicillin resistant. No Gram-negative isolates producing extended spectrum betalactamases or carbapenemases were detected

### Neonatal clinical course

Of the 14 babies born outside the metropolitan unit, the majority 11/14 (79%) remained in their regional unit of birth for on-going care. Two babies were transferred to the local metropolitan unit and one was transferred out of the LHD. We were unable to follow up babies transferred out of area, excluding mortality. Most babies with EOS are referred to one of the public facilities for management and would have been captured within our data set. Furthermore, given the high proportion of preterm babies undergoing blood culture in our district (21 to 35%), it is unlikely events were missed.

All of the 65 babies were transferred to a neonatal intensive care unit/ special care unit (NICU) but only 14/65 (22%) were admitted with a primary presumptive diagnosis of sepsis. Other common reasons for admission were prematurity and/or respiratory distress.

All babies (except one unknown) subsequently diagnosed with EOS received antibiotics. Empiric treatment was reported as intravenous penicillin and gentamicin in 61/64 cases (95%). Term infants remained on antimicrobials for a median of seven days [IQR, 7.5, 5.0 to 12.5], and remained in hospital (NICU and/or ward) for a median of 7.5 days [IQR, 7.5, 3.5 to 11.0]. Forty-one (95%) preterm babies were known to have antibiotics. Preterm babies with EOS also remained on antimicrobials for a median of seven days [IQR, 13.0, 3.3 to 16.3] and remained in hospital for a median of 19 days [IQR, 34.0, 3.8 to 37.85].

### Morbidity and mortality

Thirty-three (50%) babies had serious short-term morbidity, in particular, ventilatory support requiring neonatal intensive care. Serious, short-term morbidity occurred in 8/13 (62%) term babies with non-EOGBS sepsis and 4/10 (40%) term babies with EOGBS. It was not possible to report on long-term morbidity.

Overall, 10/65 (17%) babies with EOS died; all were preterm. The rate of preterm mortality was 1.1/1000 preterm births and rate of mortality per total live births was 0.1/1000. Gestation ranged from 24 to 34 weeks and weight from 630 to 2440 grams. Six of the 10 babies that died had early-onset *E*. *coli* infection. Mortality occurred at a median of three days after birth [range 6 hours to 44 days]. All deaths occurred at the metropolitan hospital.

## Discussion

This study provides an eleven-year, “real world” view of the epidemiology of EOS in a geographically and socially diverse setting. The EOS surveillance intensity varied with up to a two-fold difference in the proportion of term babies undergoing blood culture testing across the four largest neonatal units. Aside from the variation caused by different case mix, there are likely to be significant differences in clinician thresholds for blood culture collection, which will impact on measured culture proven EOS rates. Blood culture utilisation should be included in future studies in accord with best epidemiological practice.

All-cause EOS events declined significantly with a multiplicative change in the odds of EOS reducing by approximately 9% per year over the study period. *Escherichia coli* (n = 18) and GBS (n = 18) were the most common bacteria causing EOS events. The very low frequency of EOS events limited what could be established statistically, however, the modelling showed a significant reduction in *E*. *coli* but no evidence of a significant change in EOGBS over time.

Historically, babies of ATSI mothers have been over-represented in early-onset sepsis data. In our study, there were six babies whose mothers were identified as ATSI. These babies represent 9% of all infants with EOS. This finding is similar to the proportion of ATSI women in our total cohort Table 1A & 1B and agrees with Singh et al whose work suggests the rate of EOS in babies of ATSI mothers may be decreasing [5].

The estimated incidence in 2016 of all-cause EOS in term babies was 0.19/1000 live births (95% CI, 0 to 0.4) at the overall median birth weight of 3.42 kg. After adjustment was made for gestation, birth weight, positive maternal GBS, ROM ≥18 hours and maternal age, this represented a non-significant decline from 0.5/1000 live births (95% CI, 0.02 to 0.97) in 2006 Fig 2. This result is consistent with other studies [5, 6].

As expected, EOS was highest in preterm infants with two-thirds of events occurring in this group. All-cause EOS events in preterm babies showed a significant decline over the years. Adjusted rates were 3.4/1000 live births in 2006 (95% CI, 0.11 to 6.76) to 1.35/1000 live births in 2016 (95% CI,-0.7 to 2.78). The most dramatic reduction occurred in babies <30 weeks gestation. After adjustment, rates in this group fell from 22/1000 live births (95% CI, 1.3 to 42.7) in 2006 to 8.8/ 1000 (95% CI, -0.5 to 18) in 2016. Preterm events that occurred from 2014–2016 were confined to babies of gestation <30 weeks Fig 2.

The decline in incidence of all preterm EOS events possibly represents the effect of local changes in the management of preterm birth which, since 2011, has included routine pre-emptive antibiotic treatment upon presentation of mothers in preterm, or suspected preterm labour (Murray, H., personal communication, 2018) and advances in neonatal intensive care provision over the study period.

A universal screening approach was recommended in our LHD across the study years, with risk factors considered if a woman was GBS unknown. The crude incidence of EOGBS across term and preterm groups in our study was low at 0.19/1000 live births and, despite minor fluctuations, did not change significantly over time. Whilst we acknowledge that EOGBS rates have increased and decreased across various jurisdictions during our study period, our results compare favourably with the contemporary incidence of EOGBS recorded by the English Neonatal Infection Surveillance unit, 0.57/1000 live births, using a risk factor approach [23]; a large, multi-centre study from the US, 0.2/1000 live births, using a universal screening approach [5] and 0.33/1000 live births in a recently published Australasian study [17]. In Australia either a universal or risk factor approach is used. New Zealand recommends a risk-based approach [24]

The use of IAP in our cohort has not been associated with a detectable increase in antimicrobial resistance or EOS due to non-GBS causes. However, increasing community colonisation with extended spectrum betalactamase-producing *E*. *coli* (7.5% in 2015) is documented in Australia and may, in future lead to increasing EOS events due to such strains [25] Table 2. In the tertiary facility, there have been two multi-resistant *E*. *coli* EOS events subsequent to this study’s time period with isolates that were both resistant to gentamicin. One isolate was also resistant to ceftriaxone (*CTX-M-1* extended spectrum betalactamase gene detected).

Limitations of this study include the focus on reported culture-proven EOS events, which may have under-estimated the true burden of EOS due to false negative culture results. While our models adjusted for relevant variables, we were unable to use all EOS risk factors, as history of GBS bacteriuria and having a previous child with EOGBS were not available from the database. Fever is currently stated as a risk factor for EOS but was poorly documented and therefore not used in our analysis.

The observational design may limit generalisability and is unable to rule out all possible biases. Our study is however, generalisable to other diverse jurisdictions that can link pathology data with a maternity outcomes database such as ObstetriX or e. Maternity.

We believe strengths of this study are the inclusion of women with low and high-risk pregnancies across a geographically and socially diverse region, over a long period of time, in a setting where maternal universal screening for GBS was in place throughout the study period. Further, unique to our study, the inclusion, and evaluation of EOS surveillance intensity by studying blood culture utilisation.

### Future direction

Neonatal sepsis is an important issue in both low and high-income countries. At best, these invasive infections separate mothers and babies and strongly impact on the health care budget. At worst, invasive sepsis increases preterm birth, stillbirth, neonatal morbidity, and mortality. Although rates of GBS, the most common bacteria affecting term babies, remained low throughout our study, the potential for a vaccination to reduce risk of EOS due to GBS is attractive. In a recent multi-centre trial, GBS CPS III-TT conjugate vaccine significantly delayed maternal vaginal and rectal GBS serotype III colonisation [26].

However, a significant proportion of EOS occurs before a pregnancy is term and is due mainly to non-GBS causes. Most preterm babies do not benefit from maternal third trimester GBS screening and may not benefit from GBS vaccination. Whilst antenatal and intrapartum vigilance for signs of sepsis is crucial, a more upstream prevention such as optimisation of maternal factors that influence the neonatal and vaginal microbiome is worth consideration.

Finally, a tool that can assist clinicians in predicting the probability of EOS based on maternal risk factors as well as the baby’s clinical presentation is being used in West Australia. Using this tool, the risk of EOS can be calculated in a baby born ≥34 weeks gestation [27]. The interactive calculator produces the probability of EOS per 1000 births. As well as reducing the proportion of newborns undergoing laboratory testing and receiving empirical antibiotic treatment [28], clinical care algorithms can assist a family’s decision making around GBS screening and IAP provision, thereby possibly reducing the number of women and babies unnecessarily exposed to this intervention[29]. Such public health initiatives are best implemented, guided and audited by a multi-disciplinary maternity and neonatal care team.
